# Determination of Cytisine and N-Methylcytisine from Selected Plant Extracts by High-Performance Liquid Chromatography and Comparison of Their Cytotoxic Activity

**DOI:** 10.3390/toxins12090557

**Published:** 2020-08-29

**Authors:** Anna Petruczynik, Karol Wróblewski, Justyna Misiurek, Tomasz Plech, Karolina Szalast, Krzysztof Wojtanowski, Tomasz Mroczek, Grażyna Szymczak, Monika Waksmundzka-Hajnos, Piotr Tutka

**Affiliations:** 1 Department of Inorganic Chemistry, Medical University of Lublin, Chodźki 4a, 20-093 Lublin, Poland; justyna.misiurek@umlub.pl (J.M.); monika.hajnos@umlub.pl (M.W.-H.); 2 Department of Experimental and Clinical Pharmacology, University of Rzeszów, Kopisto 2a, 35-959 Rzeszów, Poland; tutka@umlub.pl; 3 Laboratory for Innovative Research in Pharmacology, University of Rzeszów, Kopisto 2a, 35-959 Rzeszów, Poland; 4 Department of Pharmacology, Medical University of Lublin, Chodźki 4a, 20-093 Lublin, Poland; Tomasz.plech@umlub.pl (T.P.); karolina.szalast@umlub.pl (K.S.); 5 Department of Pharmacognosy with Medicinal Plant Unit, Medical University of Lublin, Chodźki 1, 20-093 Lublin, Poland; krzysztof.wojtanowski@umlub.pl (K.W.); tomasz.mroczek@umlub.pl (T.M.); 6 Botanical Garden of Maria Curie-Skłodowska University in Lublin, Sławinkowska 3, 20-810 Lublin, Poland; grazyna.szymczak@poczta.umcs.lublin.pl; 7 National Drug and Alcohol Research Centre, University of New South Wales, Sydney, NSW 2031, Australia

**Keywords:** cytisine, N-methylcytisine, HPLC, plant extracts, cytotoxicity

## Abstract

Quinolizidine alkaloids exhibit various forms of biological activity. A lot of them were found in the *Leguminosae* family, including *Laburnum* and *Genista*. The aim of the study was the optimization of a chromatographic system for the analysis of cytisine and N-methylcytisine in various plant extracts as well as an investigation of the cytotoxic activities of selected alkaloids and plant extracts obtained from *Laburnum anagyroides, Laburnum anagyroides L. quercifolium, Laburnum alpinum, Laburnum watereri, Genista germanica*, and *Genista tinctoria* against various cancer cell lines. The determination of investigated compounds was performed by High Performance Liquid Chromatography with Diode Array Detection (HPLC-DAD), while High Performance Liquid Chromatography coupled with Quadrupole Time-of-Flight–Mass Spectrometry (HPLC-QTOF-MS) was applied for the qualitative analysis of plant extracts. The retention, separation selectivity, peaks shape, and systems efficiency obtained for cytisine and N-methylcytisine in different chromatographic systems were compared. The application of columns with alkylbonded and phenyl stationary phases led to a very weak retention of cytisine and N-methylcytisine, even when the mobile phases containing only 5% of organic modifiers were used. The strongest retention was observed when hydrophilic interaction chromatography (HILIC) or especially when ion exchange chromatography (IEC) were applied. The most optimal system in terms of alkaloid retention, peak shape, and system efficiency containing an strong cation exchange (SCX) stationary phase and a mobile phase consisted of 25% acetonitrile and formic buffer at pH 4.0 was applied for investigating alkaloids analysis in plant extracts. Cytotoxic properties of the investigated plant extracts as well as cytisine and N-methylcytisine were examined using human tongue squamous carcinoma cells (SCC-25), human pharyngeal squamous carcinoma cells (FaDu), human triple-negative breast adenocarcinoma cell line (MDA-MB-231), and human breast adenocarcinoma cell line (MCF-7). The highest cytotoxic activity against FaDu, MCF-7, and MDA-MB cancer cell lines was observed after applying the *Genista germanica* leaves extract. In contrast, the highest cytotoxic activity against SCC-25 cell line was obtained after treating with the seed extract of *Laburnum watereri*. The investigated plant extracts exhibit significant cytotoxicity against the tested human cancer cell lines and seem to be promising for further research on its anticancer activity.

## 1. Introduction

Alkaloids are nitrogen-containing organic compounds found in many plants, rarely animals, microorganisms, and fungi. Cytisine (1, [(1R,5S)-1,2,3,4,5,6-hexahydro-1,5-methano-8H-pyrido(1,2a)(1,5) diazocin-8-one]) and N-methylcytisine ([(−)-1,2,3,4,5,6-hexahydro-3-methyl-1,5-methano-8H-pyrido(1,2-a)(1,5)diazocin-8-one]) are quinolizidine alkaloids exhibiting various biological activity [1]. Cytisine is an α4β2 nicotinic acetylcholine receptor partial agonist, occurring in a various plants. For centuries, cytisine has been used in traditional medicine in the treatment of, e.g., migraine, insomnia, asthma, or cough. It has a chemical structure and a mechanism of action similar to nicotine. However, the peripheral effects of cytisine (e.g., on the cardiovascular system) are weaker than those of nicotine. Its effectiveness, demonstrated safety, and low price make cytisine a natural alternative to other medicines for smoking cessation [1,2]. This alkaloid is also able to cross the blood–brain barrier. Cytisine has an effect comparable to that of nicotine in stimulating dopamine release from striatal synaptosomes [3]. These properties are very promising for the development of new drugs, which could be used for the potential treatment of central nervous system disorders. Cytisine derivatives have been explored as potential drugs against Alzheimer’s and Parkinson’s diseases as well as attention deficit hyperactivity disorder 2012 [4]. García-García et al. studied in vitro acetylcholinesterase inhibition by cytisine derivatives [5]. Some studies have suggested that targeting the nicotinic acetylcholine receptors holds promise as a new therapeutic approach for the treatment of depression [6]. It was found that cytisine induces the apoptosis of HepG2 cells [7] and has anti-tumor effects on lung cancer cells by modulating reactive oxygen species-mediated signaling pathways [8]. Przybył et al. investigated the antiproliferative activities of cytisine derivatives against selected cancer cell lines [9]. They exhibit lower cytotoxicity against normal murine fibroblasts than cisplatin, which is the commonly used anticancer drug. N-(4-iodobenzyl)cytisine showed the strongest antiproliferative activity against lung (NCI-H358) and neuroepithelioma (SK-N-MC; IC50 below 10 mM) cancer cell lines. Tsypysheva et al. also investigated the cytotoxic properties of cytisine derivatives against cell lines НЕК293, Jurkat, A549, MCF-7, and SH-SY5Y [10]. Cytisine was nontoxic against normal human fibroblasts (BJ), human squamous cell carcinoma (SCC-15), and U-118 human glioma cells up to 500 µM after 24 h incubation [11].

Cytisine was found in the seeds and many other parts of plants of the Leguminosae (Fabaceae) family, including Laburnum (*Laburnum anagyroides* = *Cytisus laburnum*, Golden Rain), Sophora (*Sophora tetraptera*), Anagyris, Baptisia, Genista, Retama, Thermopsis, and Ulex spp. [12,13].

Some methods have been published for the chromatographic determination of cytisine in various samples. Most HPLC analyses were performed on C18, sometimes C8, columns with mobile phases containing most often acetonitrile and rarely methanol organic modifiers as well as the addition of acids and acidic buffers but rarely salts. In most described procedures, HPLC was coupled to mass spectrometry (MS) or tandem mass spectrometry (MS/MS), while UV-Vis detection was rarely applied for the determination of cytisine. For example, 22 toxic plant alkaloids, including cytisine in herbal and urine samples, were analyzed by LC-MS/MS [14]. Separation was performed on a C8 column with a mobile phase containing acetonitrile, water, and formic acid. Cytisine, matrine, and oxymatrine in radix *Sophorae tonkinensis* extracts were determined simultaneously on a C18 column with a mobile phase containing acetonitrile, water, and phosphoric acid [15]. Thirteen plant alkaloids including cytisine in a human specimen such as serum or urine were determined on a C18 column with a mobile phase containing acetonitrile and phosphate buffer at pH 6.5 [16]. Jeong et al. investigated the pharmacokinetics of cytisine in healthy smokers after a single-dose administration over a 24 h period [17]. Chromatographic separation was performed on a C18 column with a mixture of acetonitrile and ammonium formate buffer at pH 4.5. Cytisine in various body fluids and tissues after intoxication by tea prepared from the plant material of *Laburnum anagyroides* was determined on a C18 column with a mobile phase containing acetonitrile, water, and ammonium formate [18].

Ultra-performance liquid chromatography coupled to mass spectrometry (UHPLC-MS/MS) was also applied for the analysis of cytisine. For example, Zhang et al. investigated the influence of different processing methods on the oral toxicity of *Sophora alopecuroides* L. seeds in mice and on the contents of five known toxic-effective quinolizidine alkaloids including cytisine in plant extracts [19]. The alkaloids were determined by UHPLC-MS/MS using a C18 column and a mixture of acetonitrile, water, acetic acid, and ammonium acetate. UHPLC-MS/MS was also applied for the analysis of 34 toxic principles of plant origin including cytisine [20]. Analytes were separated on a C18 column with a mixture of acetonitrile and ammonium formate buffer at pH = 3.0. Cytisine and other quinolizidine alkaloids from *Sophora alopecuroides* seeds were determined by UHPLC-MS/MS on a C18 column [19,21]. Mobile phases consisting of acetonitrile and acetate buffer [19] or acetonitrile, ammonia, and ammonium acetate [21] were applied.

All the described procedures applied chromatographic analysis in reverse phase (RP) using alkyl stationary phases (C18 or C8) and aqueous-organic mobile phases with the addition of acids or buffers at acidic pH. Cytisine, a polar compound with low mass, in these chromatographic systems has a retention time close to the solvent front. It is a main disadvantage in the analysis of cytisine in RP-HPLC systems. An alternative solution to the problem in the analysis of cytisine is the application of other chromatographic methods, e.g., hydrophilic interaction chromatography (HILIC) or ion exchange chromatography (IEC). Wang et al. for the analysis of cytisine and other quinolizidine alkaloids from *Sophora alopecuroides* L. seeds applied the hydrophilic interaction chromatography (HILIC) technique that uses a polar stationary phases such as silica or a polar bonded phases in conjuction with a mobile phase containing an appreciable quantity of water combined with a higher proportion of a less polar solvent (often acetonitrile). In the procedure, analytes were separated on an amide column with a mobile phase containing acetonitrile, water, formic acid, and ammonium acetate [22].

In our previous investigations, we examined various chromatographic systems for the analysis of cytisine in serum, saliva, and pharmaceutical formulation [23]. The aim of this work was the optimization of a chromatographic system for the analysis of cytisine and N-methylcytisine in various plant extracts. The separation and quantification of these alkaloids in plant extracts were performed on an SCX column with mobile phases containing acetonitrile and formate buffer at pH 4.0. The application of IEC allowed obtaining a significantly stronger retention of the investigated compounds compared to the previously used RP systems. The applied system also lets us obtain very symmetrical peaks and high system efficiency. Cytotoxic activities of plant extracts obtained from *Laburnum anagyroides, Laburnum anagyroides quercifolium, Laburnum alpinum, Laburnum watereri, Genista germanica L.,* and *Genista tinctoria* were also investigated against various cancer cell lines.

## 2. Results and Discussion

### 2.1. Optimization of Chromatographic System

Cytisine and N-methylcytisine standards were chromatographed using various stationary phases and eluent systems for choose the optimal chromatographic conditions for their analysis in plant extracts. For this purpose, retention, separation selectivity, peaks shape, and systems efficiency obtained for both alkaloids in various chromatographic systems were compared (Table 1). The major problem with the analysis of alkaloids is that the interaction of basic alkaloids with residual silanol functional groups results in strong peak tailing and decreasing system efficiency. The application of acidic or basic buffer solutions, ion pair reagents, and free silanol blocking reagents was commonly applied to solve the problem. The choice of kind of column and composition of mobile phase is a key factor in obtaining optimal chromatographic parameters for the analysis of investigated compounds especially in complex matrices such as plant extracts.

Initially, the experiments were carried out on a Hydro RP column with an alkyl-bonded stationary phase used most often. Cytisine and N-methylcytisine were weakly retained on the stationary phase despite the application of mobile phases containing only 5% of acetonitrile in aqueous mobile phases containing various additives. In most applied mobile phases (with the addition of diethylamine (DEA), NaPF_6_, 1-butyl-3-methylimidazolium tetrafluoroborate (IL BF_4_) and 1-butyl-3-methylimidazolium hexafluorophosphate (IL PF_6_)), very asymmetrical peaks and low efficiency were obtained. Only in a system containing 5% MeCN, water and 0.025 ML^−1^ NaBF_4_ the investigated compounds were more strongly retained (t_R_ about 10 min), and symmetrical peaks (As = 0.82 and 0.84) and high N/m (45,000 for cytisine and 39,000 for N-methylcytisine) were obtained as well. However, the chromatographic system was not suitable for the analysis of the alklaoids due to the poor selectivity of their separation (t_R_ = 10.10 min for cytisine and 10.36 min for N-methylcytisine). 

For this reason, in the next steps of the experiments, analyses were performed on Phenyl-Hexyl and Polar RP columns; the phenyl bonded stationary phases were dedicated mainly for basic analytes. However, the application of a Phenyl-Hexyl column did not result in an improvement of the chromatographic parameters in this case. Investigated alkaloids were still weakly retained in most eluent systems, asymmetrical peaks were obtained, and system efficiencies were low. A better shape of peaks was observed only in a system with a mobile phase containing an addition of DEA, but the analytes were practically eluted together. 

The application of a Polar RP column resulted in an increase of alkaloids’ retention in all tested eluent systems. On the column, in most of the investigated mobile phases, the tailing of peaks was observed, and systems efficiency was low. The relatively best results were in a system with a mobile phase containing 5% MeCN, water, and 0.025 ML^−1^IL PF_6_. Analytes were well separated and symmetrical peaks were obtained, but N/m values were only about 10,000.

Due to a very weak retention, asymmetrical peaks, and poor system efficiency on octadecyl and phenyl stationary phases in most mobile phases systems, the next experiments were performed in HILIC mode. The retention mechanism in HILIC is a combination of various interactions: hydrophilic, ion exchange, and typically for reverse-phase hydrophobic interaction. To select optimal conditions, three columns with different properties were applied for the simultaneous analysis of cytisine and N-methylcytisine: ACE HILIC-A with a silica stationary phase, ACE HILIC-B with aminopropyl, and ACE HILIC-N with a polyhydroxy stationary phase. On all HILIC columns, a mixture containing 90% MeCN and formic buffer at pH 4.0 as a mobile phase was applied. The differences in retention, separation selectivity, peaks symmetry, and systems efficiency were observed for the investigated alkaloids on various HILIC columns (Table 1). On the HILIC B column, alkaloids were weakly retained, and peaks were very asymmetrical. Better results were obtained on the other HILIC columns. Both analytes were fully separated on the HILIC A and HILIC N columns. Obtained peaks were symmetrical for both alkaloids, especially on the HILIC A column, the symmetry of peaks was proper (As = 1.09 for cytisine and 1.39 for N-methylcytisine). N/m values obtained on the HILIC A column were also high (39,570 for cytisine and 37,620 for N-methylcytisine). However, the HILIC A column was not selected for the analysis of investigated compounds in plant extracts, because the peak of N-methylcytisine was eluted with the other components of the investigated extracts in this system. 

Further optimization was performed on an SCX column. A mixture of 25% MeCN and formic buffer at pH 4.0 was selected for the analysis of investigated alkaloids. In the chromatographic system, the full separation of both determined alkaloids and also the other components of plant extracts was achieved. Peaks obtained for both compounds were symmetrical (As = 1.15 for cytisine and 1.38 for N-methylcytisine). The application of the SCX column leads to obtaining the highest system efficiency from all the investigated systems (N/m were 55,000 for cytisine and 46,200 for N-methylcytisine). Based on the obtained results considering retention, separation selectivity, peak shape, and system efficiency, the determination of cytisine and N-methylcytisine in plant extracts was performed on the SCX column with the mobile phase containing 25% of MeCN and 100 mM of formate buffer at pH 4.0.

### 2.2. Determination of Cytisine and N-methylcytisine in Plant Extracts

For the extraction of cytisine and N-methylcytisine from plant materials, two procedures of extraction were applied. Both procedures were based on those early described after appropriate modifications [24]. The most important difference in procedure II compared to procedure I was the addition of KOH to ethanol in the first step of extraction. 

The identities of the analyte peaks in the plant extracts were confirmed by the comparison of their retention times and UV spectra with the retention times and spectra of alkaloid standards. For the determination of cytisine and N-methylcytisine extracts obtained from the leaves of *Laburnum anagyroides, Laburnum alpinum, Laburnum watereri, Laburnum anagyroides* L. *quercifolium*, and *Genista germanica L.* cortex of *Laburnum anagyroides*, herb of *Genista tinctoria* and seeds of *Laburnum anagyroides* and *Laburnum watereri* were analyzed by HPLC. Cytisine and N-methylcytisine were identified in most of the investigated plant extracts. Great differences in the alkaloid’s contents were obtained in extracts from various plant species and different parts of the same plant (Table 2). The content of alkaloids in extracts obtained by two extraction procedures was also compared. For extracts obtained by two compared extraction procedures, great differences in the alkaloid’s contents were noticed. Extraction procedure II was definitely better for the extraction of cytisine from almost all of the investigated plant extracts. For example, the content of cytisine in the extract obtained by procedure II from *Laburnum alpinum* leaves was 1.543 mg mL^−1^, while in the extract obtained by procedure I, it was only 0.487 mg mL^−1^. Similarly, in extracts obtained by procedure II from *Laburnum watereri* leaves, it was 0.679 mg mL^−1^, but by procedure I, it was 0.166 mg mL^−1^. Only in extracts obtained from herb of *Genista tinctoria* by both extraction procedures did we find similar contents of cytisine, but the cytisine contents in these extracts were low. Different extraction yields by two procedures were obtained for N-methylcytisine. A higher content of the alkaloid was obtained for most extracts when procedure I was applied. For example, in extract from *Genista tinctoria* herb obtained by procedure I, 0.400 mg mL^−1^ of N-methylcytisine was determined, while in the extract from the same plant material obtained by procedure II, 0.189 mg mL^−1^ was found. For extracts with low contents of N-methylcytisine, similar results were obtained by two procedures. The highest content of cytisine was obtained in the extract from *Laburnum watereri* seeds and *Laburnum alpinum* leaves (1.543 mg mL^−1^ in both extracts). High contents of the alkaloid were also determined in the extracts obtained from *Laburnum anagyroides* L. *quercifolium* leaves, *Laburnum anagyroides* leaves, and *Laburnum watereri* leaves (0.993, 0.679, and 0.679 mg mL^−1^ respectively). The lowest content of cytisine was determined in the extract obtained from *Genista tinctoria* herb. The highest content of N-methylcytisine was determined in the extract obtained from herb of *Genista tinctoria* (0.400 mg mL^−1^). A high content of the alkaloid was also found in extract obtained from *Laburnum alpinum* leaves (0.299 mg mL^−1^). 

Great differences in the contents of the investigated alkaloids have been found not only in various plant species and also in different parts of the same plant species. Contents of cytisine and N-methylcytisine were compared in extracts obtained from *Laburnum anagyroides* leaves, cortex, and seeds, and from *Laburnum watereri* leaves and seeds. Among the three extracts obtained from *Laburnum anagyroides*, the highest concentration of cytisine was determined in extract obtained from seeds (0.993 mg mL^−1^), while the lowest concentration of cytisine was determined in extract obtained from the cortex (0.228 mg mL^−1^). Similar results were obtained for the extracts from *Laburnum watereri.* In extract from seeds of the species, a very high content of cytisine was found (1.543 mg mL^−1^), while in extract from the leaves, 0.679 mg mL^−1^ of the alkaloid was determined. The results indicate an accumulation of cytisine in the seeds of these plant species. Different results were observed for N-methylcytisine. A higher content of the alkaloid was determined in the leaves and cortex, while in the seeds of both plant species, the contents of N-methylcytisine were very low (only 0.009 mg mL^−1^ and 0.018 mg mL^−1^ in seeds of *Laburnum anagyroides* and *Laburnum watereri*, respectively). 

**Figure 1:**
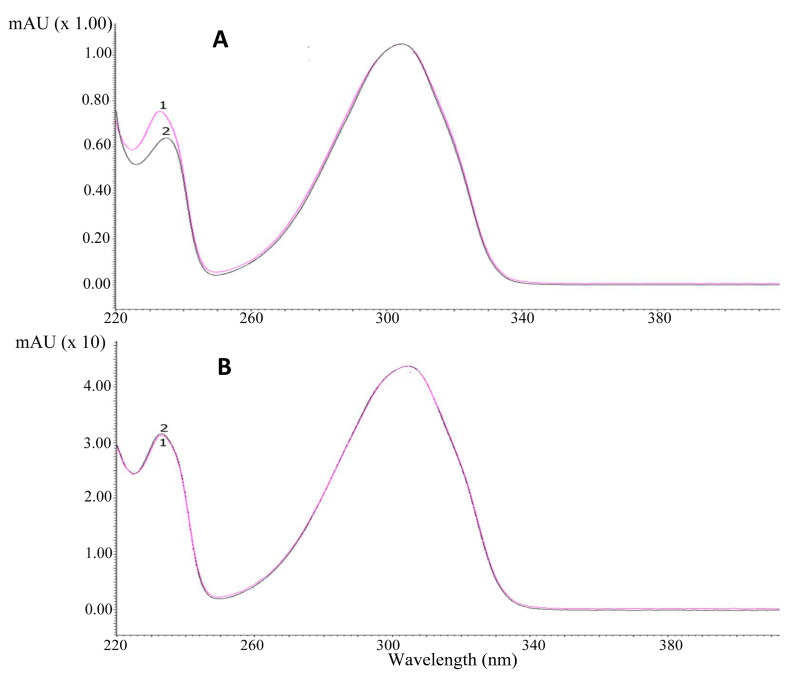
UV-Vis spectra of (**A**) cytisine, (**B**) N-methylcytisine. Spectrum 1 was obtained for alkaloid standards (marked in pink), and Spectrum 2 was obtained for alkaloids from *Laburnum anagyroides* seed extract (marked in black).

**Figure 2:**
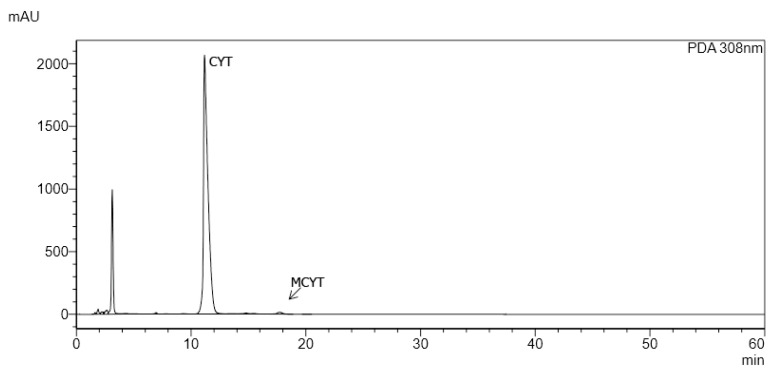
HPLC-DAD chromatogram obtained for extracts from seeds of *Laburnum anagyroides.* CYT: cytisine, MCYT: N-methylcytisin.

Identification of the investigated alkaloids was performed by comparison of their retention time and UV spectra in plant extracts (marked in black) with the spectra of standards (marked in pink) (Figure 1A,B). Examples of chromatograms obtained by HPLC-DAD are presented in Figure 2.

**Figure 3:**
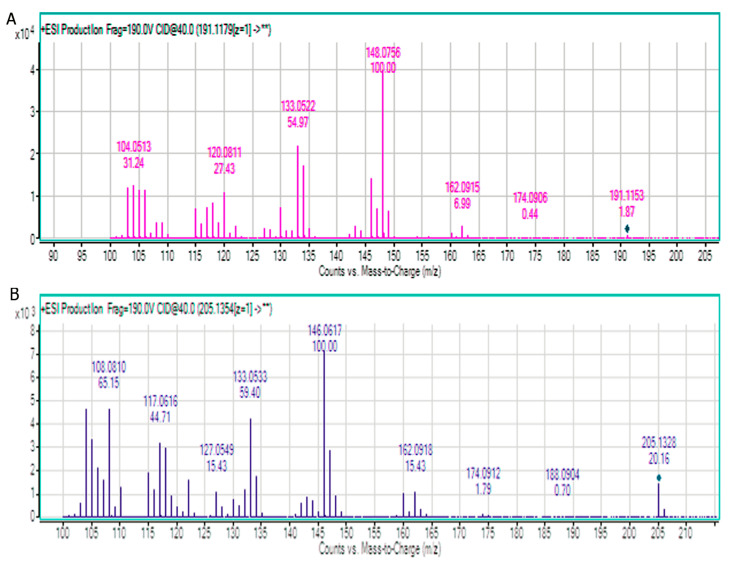
MS spectra obtained for standards of (**A**) cytisine and (**B**) N-methylcytisine.

The detection of cytisine and N-methylcytisine in extracts was also performed by LC-QTOF-MS. After chromatographic system optimization, an HILIC stationary phase with gradient elution mode (acetonitrile with 0.2% HCOOH and 50% acetonitrile in water with 0.2% HCOOH) was selected as the most optimal for investigated alkaloids analysis. MS spectra obtained for the standards of alkaloids are presented in Figure 3.

**Figure 4:**
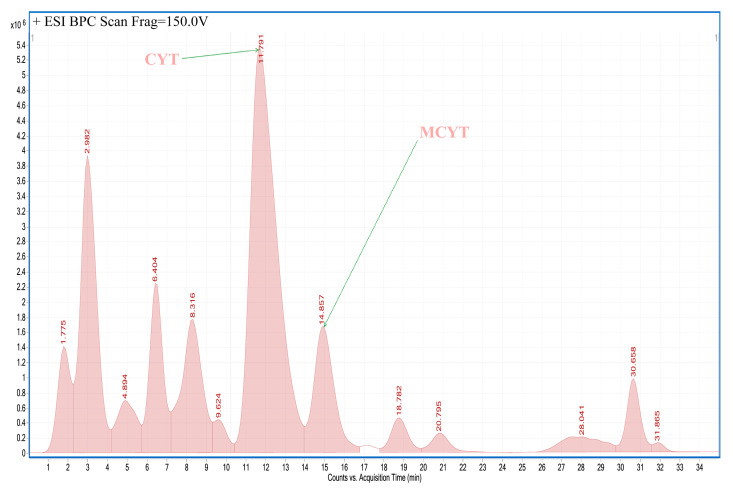
LC-MS chromatogram obtained for extract from seeds of *Laburnum anagyroides.* CYT: cytisine, MCYT: N-methylcytisine.

**Figure 5:**
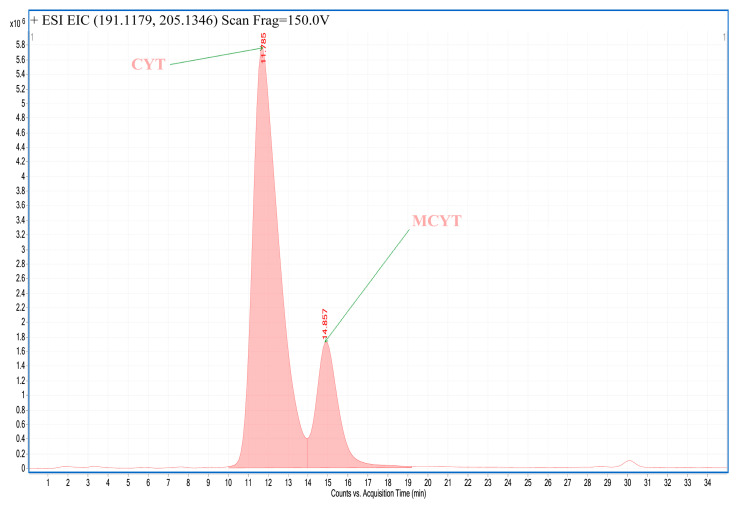
Extracted ion chromatogram obtained for extract from seeds of *Laburnum anagyroides.* CYT: cytisine, MCYT: N-methylcytisine.

An example of an LC-MS chromatogram obtained for plant extracts is presented in Figure 4. An example of an extracted ion chromatogram obtained for investigated alkaloids detected in extacts is presented in Figure 5. 

### 2.3. Cytotoxic Activity

In the next step of experiments, the cytotoxic activity of cytisine, N-methylcytisine, and plant extracts against human pharyngeal squamous carcinoma cells (FaDu), human tongue squamous carcinoma cells (SCC-25), human breast adenocarcinoma cell line (MCF-7), and human triple-negative breast adenocarcinoma cell line (MDA-MB-231) were investigated. 

The cells were treated by all plant extracts (obtained according to the most optimal procedure II) in concentrations of 10, 25, 50, and 100 µg mL^−1^. Results were reported as the percent growth of the treated cells when compared to the untreated control cells (Table 3). The application of plant extracts in concentrations of 10 µg mL^−1^ did not reduce the viability of cells belonging to all tested lines below 60%. Almost all cell lines treated by all plant extracts exhibited viability over 50% except for SCC−25 cell line treated by *Laburnum watereri* seeds extract (viability 25%). The increase in plant extracts concentration to 50 µg mL^−1^ resulted in a significant decrease of cells viability in many cases. For example, the viability of SCC-25 cells treated by *Laburnum anagyroides* leaves extract at a concentration of 25 µg mL^−1^ was higher than 60%, while at a concentration of 50 µg mL^−1^, it was 4.6%. The viability of MCF-7 cells were 97.76% and 1.03% after the application of extract from *Genista germanica* leaves at concentration of 25 and 50 µg mL^−1^, respectively. The increase in the concentration of plant extracts to 100 µg mL^−1^ enhanced their cytotoxic activity against all tested cell lines. Only after treating all of the investigated cell lines by extract obtained from leaves of *Laburnum anagyroides L. quercifolium* at a concentration of 100 µg mL^−1^ was viability over 50%. The highest cytotoxic activity against FaDu, MCF-7, and SCC-25 cell lines was observed when the cells were treated by extract from *Genista germanica* leaves. After application of the extract in a concentration of 100 µg mL^−1^, the viability of FaDu, MCF-7, and SCC-25 cells were very low, and it was only 0.16%, 0.93%, and 0.18%, respectively. The lowest viability of SCC-25 cell line was seen after the application of extract from *Laburnum watereri* seeds (only 0.64%).

The cytotoxic activity of cytisine and N-methylcytisine standards was also investigated against the same cancer cell lines as applied in investigations of the plant extracts’ cytotoxic activity. Cancer cells were treated by both alkaloid standards in concentrations from 1 to 200 µg/mL. Cytisine and N-methylcytisine practically did not exhibit antiproliferative activities against all tested human cancer cells. This indicates that the antiproliferative activity of investigated plant extracts against tested cancer cells was due to the presence of components other than cytisine and N-methylcytisine. This finding requires further research. Other alkaloids detected in some plant extracts are presented in Table 4.

## 3. Conclusions

Cytisine and N-methylcytisine are highly polar compounds and therefore are very weakly retained in most RP systems. These compounds as organic bases are strongly interacting with free silanol groups, which results in their low system efficiencies and very asymmetrical peaks on chromatograms. Only in some cases did the addition of diethylamine or some ILs result in an improvement in the shape of the peaks, but in these chromatographic systems, cytisine and N-methylcytisine were still weakly retained and not fully separated. The improvement of peak shape and system efficiency was obtained in HILIC systems using HILIC A and HILIC N columns, but in these systems, peaks of analytes were not separated from the other components of some investigated plant extracts. The strongest retention, excellent shape of the peaks, and high system efficiencies were obtained for cytisine and N-methylcytisine using the IEC method on an SCX column with mobile phases containing MeCN and formic buffer at pH 4.0. Therefore, the system was chosen for the quantification of investigated compounds in plant extracts. Most of the investigated extracts contained various quantities of cytisine and N-methylcytisine. The highest content of cytisine was determined in extracts obtained from leaves of *Laburnum alpinum* and seeds *Laburnum watereri*, while the highest contents of N-methylcytisine were found in extracts from herb of *Genista tinctoria* and leaves of *Laburnum alpinum.*

Almost all of the investigated extracts showed cytotoxic activity against tested cell lines: FaDu, SCC-25, MCF-7, and MDA-MB. The highest cytotoxic activity against FaDu, MCF-7, and MDA-MB cancer cell lines was observed after applying the *Genista germanica L*. leaves extract. A lowe viability of SCC-25 cell line was determined after treating by extract obtained from seeds of *Laburnum watereri*. The cytotoxic activity of the investigated extracts was not related to the content of cytisine and N-methylcytisine, because the standard solutions of these compounds did not show similar cytotoxicity to the tested cell lines.

## 4. Materials and Methods

### 4.1. Chemicals and Plant Material

Acetonitrile (MeCN), methanol (MeOH), 1-butyl-3-methylimidazolium tetrafluoroborate, sodium tetrafluoroborate, formic acid, and ammonium formate of chromatographic quality were obtained from E. Merck (Darmstadt, Germany), 1-butyl-3-methylimidazolium hexafluorophosphate, sodium hexafluorophosphate, dimethyl sulfoxide (DMSO) were from Sigma-Aldrich (Saint Louis, MO, USA).

The standard of cytisine was obtained from Aflofarm (Pabianice, Poland). N-methylcytisine standard was purchased from Sigma-Aldrich (St. Louis, MO, USA).

Plant material was collected and identified in the Botanical Garden of Maria Curie-Skłodowska University in Lublin (Poland) in the spring of 2019.

Plants organs were cut into pieces and dried at ambient temperature for one to two weeks.

### 4.2. Extraction Procedures

#### 4.2.1. Procedure I

First, 100 mL of ethanol was added to samples (5 g) of each plant material. The maceration time was 48 h. Next, samples were continuously extracted in an ultrasonic bath for 5 h. Obtained extracts were filtered, and the solvent was evaporated under vacuum. Then, the residues were dissolved in 2% sulfuric acid solution (30 mL) and degreased with diethyl ether (3 × 40 mL). Next, the aqueous layers were basified with 25% ammonia to obtain a pH 9.5–10. Then, the alkaloids were extracted with chloroform (3 × 50 mL). After evaporation of the organic solvent, the residues were dissolved in MeOH (5 mL). The aliquot of each obtained solution was injected directly into the HPLC column.

#### 4.2.2. Procedure II

To samples (5 g) of each plant material, 100 mL of 2% KOH in ethanol were added, and samples were extracted in an ultrasonic bath for 2 h. After filtration, samples were extracted with chloroform (3 × 25 mL), and the solvent was evaporated under vacuum. Next, the residues were dissolved in chloroform (25 mL) and 2% sulfuric acid solution (30 mL). The aqueous layers were basified with 25% ammonia to obtain a pH 9.5–10. Then, the alkaloids were extracted with chloroform (3 × 25 mL). After evaporation of the organic solvent, the residues were dissolved in MeOH (5 mL). The aliquot of each obtained solution was injected directly into the HPLC column.

### 4.3. HPLC-DAD

Various stationary phases were used for chromatographic analyses. The parameters of applied columns are presented in Table 5. The detection of cytisine and N-methylcytisine in plant extracts was conducted using an SCX column. The analyses were performed at 22 °C in isocratic mode with eluent consisting of 25% of MeCN and 100 mM of formate buffer at pH 4.0. The eluent flow rate was 1.0 mL/min. The injection volume was 20 μL. The DAD detector was set in the 200–400 nm range, and qualitative analysis was performed at 308 nm.

Calibration curves were prepared using eight concentrations of cytisine ranging from 0.025 to 1 µg mL^−1^ and seven concentrations of N-methylcytisine from 0.025 to 0.5 µg mL^−1^ in triplicate. Limit of Detection (LOD) and Limit of Quantitation (LOQ) were calculated according to the formula: LOD = 3.3 (SD/S), and LOQ = 10 (SD/S), where SD is the standard deviation of response (peak area) and S is the slope of the calibration curve.

The coefficient of correlation, slope, and intercept was calculated for linearity evaluation using the injections of above solutions in triplicate.

### 4.4. LC-MS/MS

An HPLC/ESI-QTOF-MS system was applied for the qualitative analysis of plant extracts. A normal phase (NP) Atlantis HILIC silica column (150 × 2.1 mm, dp = 3 μm) (Waters Milford, MA, USA) was applied as a stationary phase. The chromatograph was equipped with a binary gradient pump, autosampler, column oven (25 °C), and DAD detector. Acetonitrile with 0.2% HCOOH was used as mobile phase A and 50% acetonitrile in water with 0.2% HCOOH was used as mobile phase B. The following gradient was adopted: 0–20 min 30–45%B; 20–25 min 45–95%B; 25–35 min 95%B; post time 10 min. Flow rate 0.3 mL/min, injection volume 10 μL, total time of analysis 45 min. The mass spectral analyses were performed using a 630B accurate mass QTOF-MS (Agilent Technologies INc., Santa Clara, CA, USA) mass spectrometer equipped with an ESI-Jet-Stream^®^ ion source operating in positive ion mode, with the following set of operation parameters: drying gas (N_2_), temperature 300 °C, flow rate 12 L/min; nebulizer pressure 35 psi; sheath gas, temperature 350 °C, flow rate 12 L/min; fragmentor voltage 140 V; ion spray voltage 4000 V. Collision induced cell at two energies: 20 and 40 eV. Data acquisition was performed in Auto MS/MS mode at the range of 100–1000 mass units for MS and MS/MS. Mass Hunter B.07.00 software was used for data analysis.

Formula, molecular ion, and fragment ions for cytisine and N-methylcytisine are presented in Table 6.

### 4.5. Investigation of Cytotoxic Activity

The cytotoxicity of the investigated plant extracts, cytisine and N-methylcytisine, was evaluated using human tongue squamous carcinoma cells (SCC-25), human pharyngeal squamous carcinoma cells (FaDu), human triple-negative breast adenocarcinoma cell line (MDA-MB-231), and human breast adenocarcinoma cell line (MCF-7). Human normal skin fibroblasts (CRL-1634) as well as HepG2 cells were applied as reference cell lines. The above cells were cultured using the following conditions. The cultivation of SCC-25 was performed using Dulbecco’s Modified Eagle’s Medium/Nutrient Mixture F-12 Ham (DMEM/F12) with the addition of 10% fetal bovine serum, 100 U/mL of penicillin, 100 mg/mL of streptomycin, and 400 ng/mL hydrocortisone (all obtained from Sigma Aldrich). HepG2 and FaDu cells were cultured in Eagle’s Minimum Essential Medium (MEM) with the addition of 10% fetal bovine serum, 100 mg/mL of streptomycin, and 100 U/mL of penicillin. The cultivation of MDA-MB-231, MCF-7, and CRL-1634 cells was performed using high-glucose Dulbecco’s Modified Eagle’s Medium (DMEM) with the addition of 10% fetal bovine serum, 100 mg/mL of streptomycin, and 100 U/mL of penicillin. Cells were kept at a temperature of 37 °C in a 5% CO_2_ atmosphere. The alkaloid standards as well as dried plant extracts were dissolved in DMSO to obtain stock solutions at concentrations of 50 mg/mL and 250 mg/mL, respectively. On the day of the experiment, the suspension of cells (1 × 10^5^ cells/mL) in respective medium containing 10% FBS was applied to a 96-well plate at 100 μL per well. The time of incubation was 24 h. Next, the medium was removed from wells and replaced by various concentrations (from 10 to 100 µg/mL) of investigated plant extracts or alkaloid standards in medium containing 2% FBS. Control cells were cultured only using a medium containing 2% FBS. The cytotoxic properties of DMSO were also checked at concentrations present in respective dilutions of stock solutions. The concentration of DMSO in standard and extract solutions was 0.1%. This concentration does not affect the metabolic activity of the cells. After 24 h of incubation, 15 μL of MTT working solution (5 mg/mL in PBS) was added to each well. Then, the plate was incubated for 3 h. Next, 100 μL of 10% SDS solution was added to each well. Cells were incubated overnight at a temperature of 37 °C to dissolve the precipitated formazan crystals. A microplate reader (Epoch, BioTek Instruments, Inc., USA) was applied for evaluation of the concentration of the dissolved formazan. For these purpose, the absorbance at λ = 570 nm was measured. Two independent experiments were conducted in triplicate. The viability of cells incubated with alkaloid standards or plant extract was expressed as percentage of the viability of control (untreated) cells. DMSO used in the concentrations present in the dilutions of stock solutions did not influence the viability of the investigated cells.

## Figures and Tables

**Table 1:** Retention time (t_R_), asymmetry factor (As), and theoretical plate number per meter (N/m) values obtained for cytisine and N-methylcytisine on various columns with different mobile phases.

Column	Mobile Phase	Cytisine			N-methylcytisine		
		t_R_	As	N/m	t_R_	As	N/m
Hydro-RP	5% MeCN + 20% acetate buffer at pH 3.5 H_2_O + 0.025 ML^−1^ DEA	1.69	5.87	9860	4.69	3.78	16,970
	5% MeCN + H_2_O + 0.025 ML^−1^ NaBF_4_	10.10	0.82	45,000	10.36	0.84	39,040
	5% MeCN + H_2_O + 0.025 ML^−1^ NaPF_6_	2.67	*		3.05	*	
	5% MeCN + H_2_O + 0.025 ML^−1^ IL BF_4_	3.41	*		3.38	*	
	5% MeCN + H_2_O + 0.025 ML^−1^ IL PF_6_	4.36	*		11.23	0.66	3500
Phenyl-Hexyl	5% MeOH + 20% acetate buffer at pH 3.5 H_2_O + 0.025 ML^−1^ DEA	2.64	0.96	14,120	2.75	1.10	20,620
	5% MeCN + H_2_O + 0.025 ML^−1^ NaBF_4_	3.44	*		3.44	0.65	18,090
	5% MeCN + H_2_O + 0.025 ML^−1^ NaPF_6_	9.56	*		11.14	*	
	5% MeCN + H_2_O + 0.025 ML^−1^ IL BF_4_	2.53	*		2.72	1.48	1720
	5% MeCN + H_2_O + 0.025 ML^−1^ IL PF_6_	3.75	*		5.83	*	
Polar RP	5% MeCN + 20% acetate buffer at pH 3.5 H_2_O + 0.025 ML^−1^ DEA	3.49	0.63	15,430	4.32	2.02	20,130
	5% MeCN + H_2_O + 0.025ML^−1^ NaBF_4_	4.85	*		5.46	*	
	5% MeCN + H_2_O + 0.025 ML^−1^ NaPF_6_	9.93	0.73	27,270	12.46	0.79	23,300
	5% MeCN + H_2_O + 0.025 ML^−1^ IL BF_4_	5.99	*		6.91	*	
	5% MeCN + H_2_O + 0.025 ML^−1^ IL PF_6_	4.88	1.16	9960	9.22	1.18	9070
HILIC A	90% MeCN + formic buffer at pH 4.0	4.08	1.09	39,570	3.03	1.39	37,620
HILIC B	90% MeCN + formic buffer at pH 4.0	2.58	*		2.09	*	
HILIC N	90% MeCN + formic buffer at pH 4.0	8.00	1.10	4920	3.31	1.48	20,600
SCX	25% MeCN + formic buffer at pH 4.0	12.43	1.15	55,000	17.58	1.38	46,200

* fuzzy peak.

**Table 2:** Contents of alkaloids in plant samples.

Plant Material	Content of Cytisine (mg mL^−1^)		Content of N-Methylcytisine (mg mL^−1^)	
	Extraction Method I	Extraction Method II	Extraction Method I	Extraction Method II
*Laburnum anagyroides*—leaves	0.426	0.679	0.042	0.044
*Laburnum alpinum*—leaves	0.487	1.543	0.299	0.184
*Laburnum watereri*—leaves	0.166	0.679	0.113	0.071
*Laburnum anagyroides L. quercifolium*—leaves	0.178	0.436	0.070	0.006
*Laburnum anagyroides*—cortex	0.221	0.228	0.057	−
*Genista germanica L*.—leaves	0.109	0.464	0.035	−
*Genista tinctoria*—herb	0.062	0.058	0.400	0.189
*Laburnum anagyroides*—seeds	−	0.993	−	0.009
*Laburnum watereri*—seeds	−	1.543	−	0.018

− Not identified.

**Table 3:** Viability of cells treated by plant extracts.

Plant Material	Mean % of Control. FaDu, 1 × 10^5^, MTT * 24 h				Mean % of Control, MCF-7, 1 × 10^5^, MTT 24 h				Mean % of Control, MDA-MB-231, 1 × 10^5^, MTT 24 h					Mean % of Control, SCC-25, 1 × 10^5^.MTT 24 h			
	10 μg/mL	25 μg/mL	50 μg/mL	100 μg/mL	10 μg/mL	25 μg/mL	50 μg/mL	100 μg/mL	10 μg/mL	25 μg/mL	50 μg/mL	100 μg/mL	10 μg/mL		25 μg/mL	50 μg/mL	100 μg/mL
*Laburnum anagyroides*—leaves	61.63	51.20	38.98	2.10	62.94	62.81	54.78	5.37	71.19	66.03	36.04	9.20	64.61		60.83	4.61	3.30
*Laburnum alpinum*—leaves	109.94	96.18	73.31	2.29	109.85	105.41	92.68	21.16	97.15	95.96	46.25	7.55	109.59		104.90	14.42	2.87
*Laburnum watereri*—leaves	112.0	102.5	93.10	8.13	114.76	109.04	100.05	22.41	105.11	95.72	65.49	31.53	103.51		99.17	39.67	1.37
*Laburnum anagyroides L. quercifolium*—leaves	79.24	75.95	67.76	53.67	94.38	90.91	81.62	77.62	84.45	78.92	69.26	66.19	91.51		88.31	75.45	64.27
*Laburnum anagyroides*—cortex	87.94	81.75	58.21	5.24	114.74	105.43	53.15	12.15	99.54	48.63	19.43	12.44	98.18		85.87	47.43	12.46
*Genista germanica L*.—leaves	102.52	60.2	2.27	0.16	117.29	97.76	1.03	0.93	101.66	96.76	2.09	0.18	114.20		108.94	6.38	3.59
*Genista tinctoria*—herb	78.84	50.28	36.32	1.28	81.06	49.32	24.58	9.07	92.01	66.40	41.11	17.76	94.23		93.77	27.46	4.51
*Laburnum anagyroides*—seeds	94.98	87.13	78.36	8.38	103.26	95.35	85.37	63.40	98.90	90.63	61.19	39.375	101.20		58.76	4.43	2.34
*Laburnum watereri*—seeds	90.62	70.38	60.67	18.56	87.14	83.54	61.75	35.06	84.14	79.03	47.74	26.06	103.22		25.56	1.20	0.64
*Etoposide*	57.73	42.57	45.79	36.74	112.14	117.49	99.00	86.39	119.18	102.78	95.59	79.22	90.02		80.48	71.87	70.25

* MTT: 3-[4,5-dimethylthiazol-2-yl]-2,5-diphenyltratrazolium bromide.

**Table 4:** List of alkaloids identified in some plant extracts by LC-QTOF-MS.

***Genistia tinctoria* Herb Extract**					
**Compound**	**Retention Time (min)**	**Formula**	**Molecular Ion [M + H]^+^**	**Fragment Ions**	**Collision Energy (eV)**
Sparteine	4.385	C_15_H_26_N_2_	235.2181	150.1273 134.0955 100.1026	40
Isolupanine	5.392	C_15_H_24_N_2_O	249.1971	219.1834 166.1215 148.1125 134.0967 110.0965	40
N-formylcytysine	7.505	C_12_H_14_N_2_O_2_	219.1499	160.0745 146.0696 133.0718 120.0746 108.0844 104.0486	40
Lupanine	9.920	C_15_H_24_N_2_O	249.1965	231.1873 150.1278 136.1119 114.0907	40
Cytisine	10.826	C_11_H_14_N_2_O	191.1182	162.0956 148.0756 133.0516 118.0641 105.0569	40
Anagyrine	12.336	C_15_H_20_N_2_O	245.1659	162.0949 148.1118 134.0959 120.0825	40
N-methylcitisine	13.543	C_12_H_16_N_2_O	205.1341	160.0790 146.0592 133.0533 117.0589 108.0793 104.0473	40
***Laburnum anagyroides* Leaf Extract**					
**Compound**	**Retention Time (min)**	**Formula**	**Molecular Ion [M + H]^+^**	**Fragment Ions**	**Collision Energy (eV)**
Laburnamin	6.800	C_12_H_22_N_2_O	211.1821	127.1236 110.0974	20
Ammodendrin	8.191	C_12_H_20_N_2_O	209.1665	150.1261 122.0939 110.0960	40
Cytisine	12.838	C_11_H_14_N_2_O	191.1194	162.0922 148.0762 133.0525 120.0811 109.0530	40
Anagyrine	14.398	C_15_H_20_N_2_O	245.1618	162.0898 148.1116 134.0952 122.0603 110.0595	40
N-methylcitisine	15.455	C_12_H_16_N_2_O	205.1358	162.0930 146.0616 133.0532 117.0621 108.0816	40
***Laburnum anagyroides L. quercifolium* Leaf Extract**					
**Compound**	**Retention Time (min)**	**Formula**	**Molecular Ion [M + H]^+^**	**Fragment Ions**	**Collision Energy (eV)**
Laburnamin	6.951	C_12_H_22_N_2_O	211.1821	127.1231 110.0974	20
Ammodendrin	7.504	C_12_H_20_N_2_O	209.1656	150.1271 138.1270 122.0960 110.0970	40
Cytisine	12.234	C_11_H_14_N_2_O	191.1184	162.0932 148.0756 133.0525 120.0807 106.0651	40
N-methylcitisine	14.750	C_12_H_16_N_2_O	205.1344	160.0748 146.0603 133.0532 118.0786 108.0786	40
***Laburnum alpinum* Leaf Extract**					
**Compound**	**Retention Time (min)**	**Formula**	**Molecular Ion [M + H]^+^**	**Fragment Ions**	**Collision Energy (eV)**
Sparteine	5.485	C_15_H_26_N_2_	235.2189	150.1268 134.0965 100.1032	40
Ammodendrin	6.424	C_12_H_20_N_2_O	209.1660	150.1279 134.0989 122.0970 105.0701	40
Lupanine	11.372	C_15_H_24_N_2_O	249.1974	164.1123 150.1278 136.1120 114.0917	40
Cytisine	12.238	C_11_H_14_N_2_O	191.1186	162.0902 148.0761 133.0524 120.0802 104.0509	40
Epi-Baptifolin	14.643	C_15_H_20_N_2_O_2_	261.1618	164.1069 114.0921	20
N-Methylcytysine	15.751	C12H16N2O	205.1344	−	−
***Aburnum watereri* Leaf Extract**					
**Compound**	**Retention Time (min)**	**Formula**	**Molecular Ion [M + H]^+^**	**Fragment Ions**	**Collision Energy (eV)**
Ammodendrin	6.456	C_12_H_20_N_2_O	209.1670	150.1269 122.0964 105.0702	40
Cytisine	12.240	C_11_H_14_N_2_O	191.1190	162.0908 148.0761 133.0525 120.0808 104.0513	40
Epi-Baptifolin	14.790	C_15_H_20_N_2_O_2_	261.1617	164.1080 114.0921	20
N-methylcitisine	15.561	C_12_H_16_N_2_O	205.1344	160.0762 146.0610 133.0527 117.0613 108.0803	40
***Genista germanica* Leaf Extract**					
**Compound**	**Retention Time (min)**	**Formula**	**Molecular Ion [M + H]^+^**	**Fragment Ions**	**Collision Energy (eV)**
Sparteine	5.184	C_15_H_26_N_2_	235.2184	150.1287 134.0969 110.0984 100.1034	40
Lupanine	11.272	C_15_H_24_N_2_O	249.1970	231.1834 204.1424 150.1274 136.1116 114.0916	40
Cytisine	12.229	C_11_H_14_N_2_O	191.1189	162.0917 148.0757 133.0528 120.0809 118.0652 105.0588	40
Anagyrine	13.738	C_15_H_20_N_2_O	245.1659	162.0910 148.1112 134.0938 122.0585 110.0595	40
Epi-Baptifolin	14.694	C_15_H_20_N_2_O_2_	261.1619	164.1073 114.0916	40
N-Methylcytysine	15.751	C_12_H_16_N_2_O	205.1344	−	−

**Table 5:** List of tested stationary phases and their physicochemical properties.

Phase	Functional Group	Length (mm)	I.D. (mm)	Endcapped	Particle Size (μm)	Pore Size (Å)	Surface Area (m^2^/g)	Carbon Load (%)	Recommended pH Range
Synergy Polar RP	Ether-linked phenyl	150	4.6	Proprietary (polar group)	4	80	475	11	1.5–7.0
CSH Phenyl-Hexyl	Phenyl-hexyl	150	4.6	Proprietary	5	130	185	15	1.0−11.0
Synergi Hydro-RP	Octadecyl (C18)	150	4.6	Proprietary (polar group)	4	80	475	19	1.5–7.5
ACE HILIC-A	Proprietary SIL	150	4.6	NO	5	100	300	−	2.0–7.0
ACE HILIC-B	Proprietary Aminopropyl	150	4.6	NO	5	100	300	4	2.0–7.0
ACE HILIC-C	Proprietary Polyhydroxy	150	4.6	NO	5	100	300	7	2.0–7.0
Luna SCX	Benzene Sulfonic Acid	150	4.6	NO	5	100	400	0.55 Sulfur Load	2.0–7.0

**Table 6:** MS parameters for cytisine and N-methylcytisine.

Compound	Formula	Molecular Ion [M + H]^+^	Fragment Ions	Collision Energy (eV)
Cytisine	C_11_H_14_N_2_O	191.1153	162.0915 148.0756 133.0522 120.0811 104.0513	40
N-methylcitisine	C_12_H_16_N_2_O	205.1344	160.0918 146.0617 133.0533 127.0549 117.0616 108.0810 104.0503	40
